# The triathlon of magnetic actuation: Rolling, propelling, swimming with a single magnetic material

**DOI:** 10.1038/srep09364

**Published:** 2015-03-20

**Authors:** Peter J. Vach, Damien Faivre

**Affiliations:** 1 Department of Biomaterials, Max Planck Institute of Colloids and Interfaces, Science Park Golm, 14424 Potsdam, Germany

## Abstract

Magnetic actuation of microscopic devices in a liquid environment has been achieved in various ways, which can be grouped into rolling, propelling and swimming. Previous actuators were designed with a focus on one particular type of magnetic actuation. We have shown earlier that efficient magnetic propellers can be selected from randomly shaped magnetic nanostructures synthesized in solution. Here we show that these synthesized nanostructures can be used for all three types of magnetic actuation. Whereas it might not be surprising that single structures can roll in addition to propelling, swimming is unexpectedly also observed using the same material. In this case, however, the magnetically guided self-assembly of several individual particles into chain-like structures is necessary to obtain swimmers, since individual rigid nanostructures cannot swim. Interestingly, the direction of the swimming motion is not necessarily parallel to the long axis of the chain-like assembly, a finding that had been theoretically expected but experimentally not observed so far. Our findings show that the range of structures that can be effectively actuated by external magnetic fields is much broader than assumed until now. This could open up new opportunities for the design of magnetically actuated devices.

Micro- and nanorobots have been the subject of numerous studies due to their high potential for applications, ranging from microassembly and sensing to minimally invasive medicine and environmental remediation^1^,^2^,^3^,^4^. Magnetic fields can be used to “fuel” such devices and are particularly promising for the actuation of microscopic devices in a liquid environment. Several mechanisms for magnetic actuation have been proposed^5^,^6^,^7^,^8^. Gradient fields can exert forces on magnetic materials and have been used successfully to move microscopic objects^9^. Magnetic field gradients, however, decay like 1/*r*^4^, where *r* is the distance from a dipolar source of magnetic fields. Therefore, when the source of the magnetic field is far away from the devices to be actuated, it becomes favorable to use the torques exerted by homogenous magnetic fields, since these only decay like 1/*r*^3^ 6.

Exerting torques does, however, not immediately lead to movement. It is thus necessary to find a way to convert torques into effective forces. The published methods to achieve such conversion can be grouped into three categories: propelling^10^,^11^,^12^,^13^,^14^,^15^,^16^, rolling^17^,^18^,^19^,^20^,^21^ and swimming^22^,^23^,^24^,^25^,^26^,^27^,^28^,^29^,^30^. Magnetic propellers are structures, which are actuated by rotating magnetic fields. The hydrodynamic coupling between rotation and translation leads to the propeller moving through the liquid, parallel to the vector of rotation of the magnetic field^10^,^11^,^12^,^13^,^14^,^15^,^16^. Rollers are objects that move along a surface when actuated by rotating or precessing magnetic fields^17^,^18^,^19^,^20^,^21^. Swimmers are objects that change shape during one period of the actuating field, in a way that leads to a net displacement of the structure^22^,^23^,^24^,^25^,^26^,^27^,^28^,^29^,^30^. These three categories are not mutually exclusive, since structures can share attributes of more than one category. For example, most propellers are also rollers if they are close to a surface^11^.

How the application of torques can lead to translatory movement can be understood in the framework of low Reynolds number hydrodynamics. The fluid movement is then described by the Stokes equations for an incompressible Newtonian fluid:
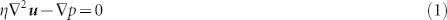

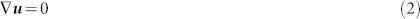
where *η* is the dynamic viscosity of the fluid, *p* is the pressure and ***u*** is the velocity of the fluid. Time does not appear as a parameter in these equations, meaning that it does not matter if a cyclic process happens slowly or fast, the net translatory movement of one cycle will be the same (Scallop theorem)^31^. Due to this fact, it is not possible to achieve translatory motion by reciprocal movement (Figure 1a)^31^. One way around this limitation is to actuate a structure in a non-reciprocal way, for example by applying a rotating magnetic field (Figure 1b). If the actuated structure is flexible, actuation can also be achieved with a field that would only induce reciprocal motion in a rigid structure (Figure 1a). Interestingly movement is also possible if a rigid structure is actuated in a reciprocal manner close to an elastic interface^32^. It should be noted that the scallop theorem does not hold in many biological liquids, which are viscoelastic media. Finally, symmetry breaking is also required. For rollers, this is achieved by being close to an interface. Propellers have to be sufficiently asymmetric in order to achieve translatory movement. Symmetry breaking for swimmers has been generally achieved using a head-tail design, where either a magnetic head is used to actuate a flexible tail, or the tail itself is magnetic^22^,^23^,^24^,^25^,^26^,^27^,^28^,^30^,^33^. These requirements for torque based translatory motion are summarized in Figure 1.

## Results and Discussion

While it is often useful to categorize microscopic actuators as propellers, rollers or swimmers, we show here that a single nanostructured material can be used to produce all three types of magnetic actuators. This material consists of carbon coated aggregates of magnetic nanoparticles (see supplementary information) of varied shapes and forms and is produced using hydrothermal carbonization^34^. The variability in this ensemble of shapes enables the versatility in magnetic actuation, but also leads different structures to move with different speeds. Due to the possibility to actuate and steer the structures, those with desirable properties can be selected in a straight-forward manner from the pool of synthesized aggregates, as we reported recently in the case of propellers^16^.

**Figure 1:**
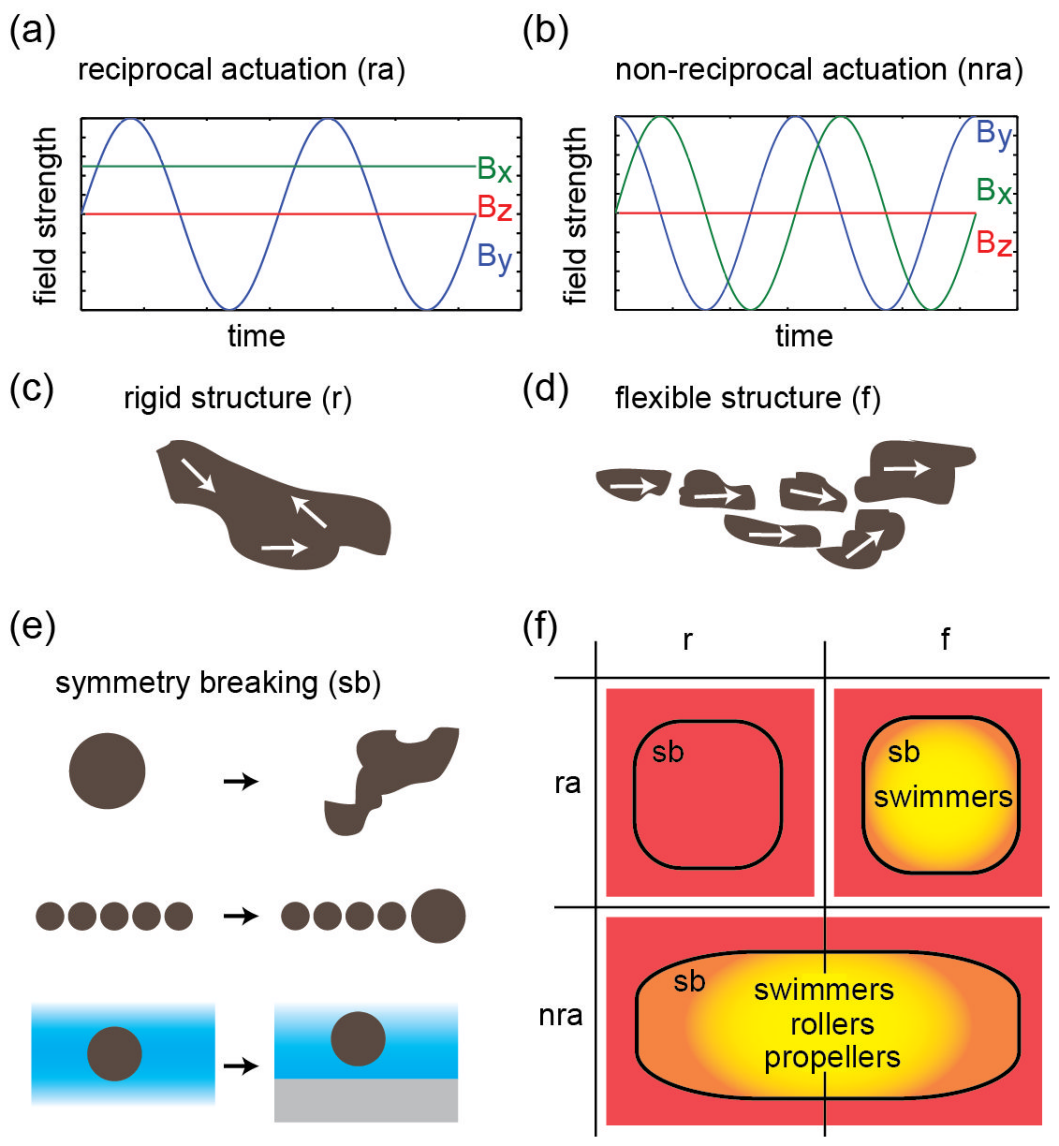
Summary of requirements for torque based translatory motion in Newtonian fluids at low Reynolds number. (a) An example of a magnetic field that will typically induce reciprocal motion in a rigid magnetic structure. The field strengths of the three components of the magnetic field vector are plotted against time. (b) The same plot as (a) for a rotating magnetic field, which induces non-reciprocal motion also in a rigid structure. (c) Schematic of a rigid magnetic structure. White arrows indicate the magnetization state. (d) Schematic of a flexible magnetic structure. The structure can incorporate physical linkers (e.g. DNA) or might be held together by magnetic interactions alone. (e) Symmetry breaking is necessary for translatory motion. This can be achieved by asymmetries in the structure itself, mixtures of different particles, or the presence of an interface. (f) The requirements are summarized in a table. Red areas mark regions where translatory motion is impossible. Yellow areas mark regions where translatory motion is possible. A gradient was used in the yellow areas to indicate that the conditions presented here are necessary, but not sufficient.

**Figure 2:**
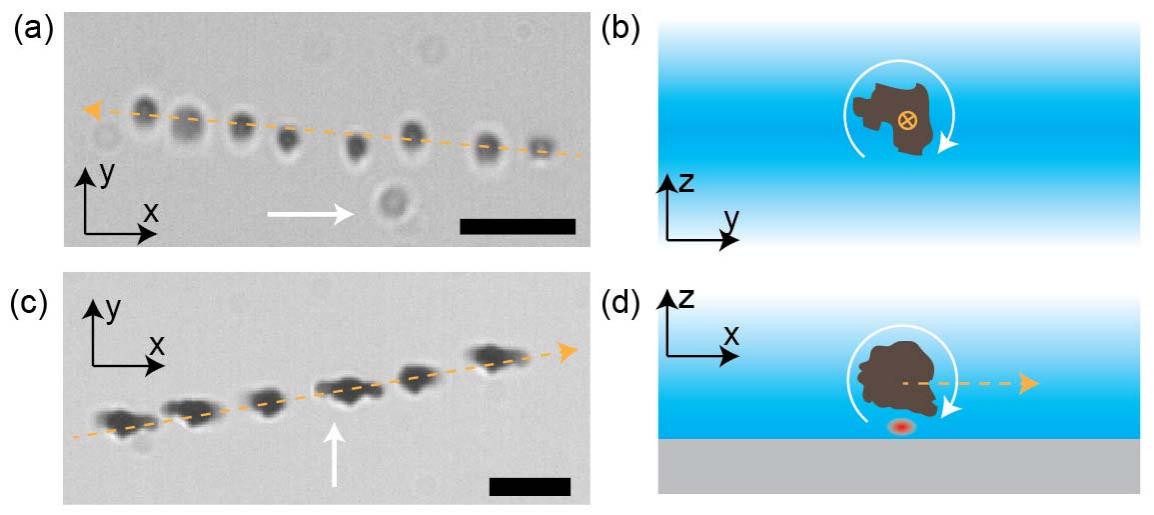
Propeller and roller from the same materials: (a) Time-lapse image of propulsion movement. A particle is rotated in bulk liquid by a rotating magnetic field. The images are overlaid in 0.52 s intervals. The x, y, z directions form a right-handed orthogonal coordinate system. (b) Schematic illustrating the propulsion mechanism. The view is rotated with respect to panel (a). Propulsion is achieved due to the hydrodynamic coupling between rotation and translation. (c) Time-lapse image of rolling movement. Images are overlaid in 0.32 s intervals. (d) Schematic illustrating the rolling mechanism. The actuated magnetic structure is pulled towards a glass surface (grey area) by gravity. The red dot marks a region in the fluid that experiences higher shear, due to the vicinity of two no-slip boundaries. The imbalance between shear forces below and above the propeller leads to translation. Images are cut from videos recorded at 50 fps. The white arrows indicate the vectors of rotation of the rotating magnetic fields (right hand rule). The dashed orange arrows indicate the directions of motion. Scale bars are 5 μm.

**Figure 3:**
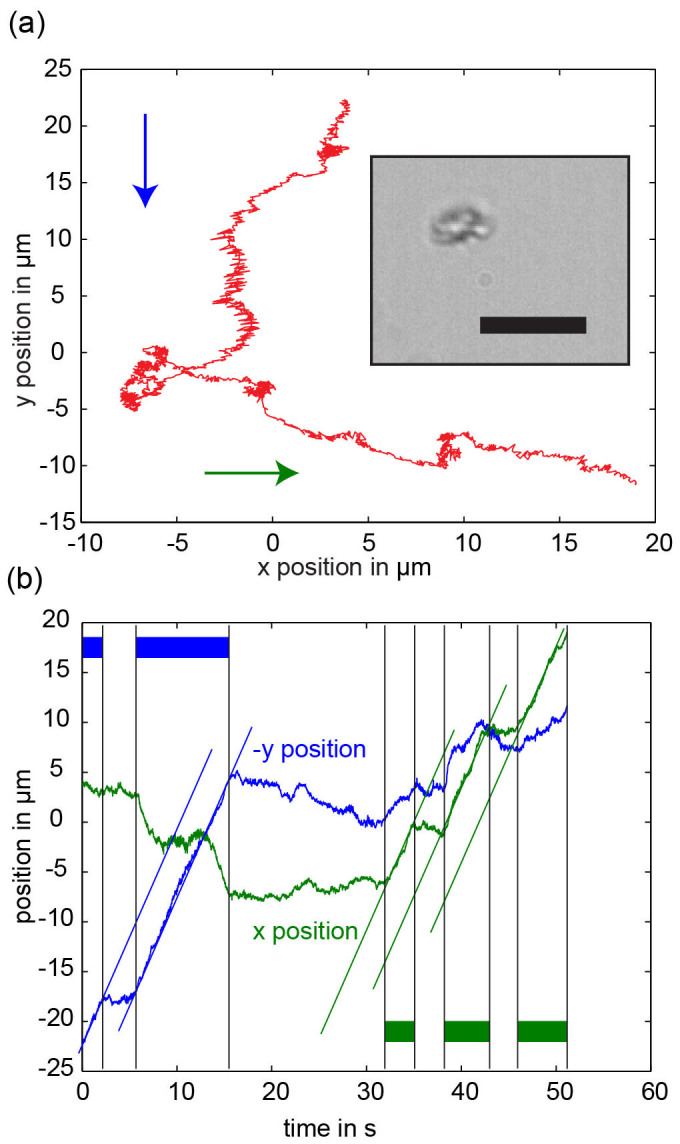
Control of the trajectory of a structure, which is simultaneously rolling and propelling: (a) a magnetic nanostructure is actuated with a rotating magnetic field (2 mT, 20 Hz). The vector of rotation is at first parallel to the y axis (blue arrow) and later parallel to the x axis (green arrow). The inset shows an optical microscopy image of the propeller. Scale bar is 5 μm. (b) The x and negative y positions of the nanostructure are plotted against time. The green and blue bars mark approximate time periods during which a rotating field is applied either with the vector of rotation along the x (green) or the y (blue) direction. The field switches on and off due to the fact that the control software tries to adjust the field strength and frequency to the correct values. The blue and green lines are all parallel to each other, showing that the propulsion proceeds with constant speed in both directions. The rolling speed (the rate of change of the coordinate orthogonal to the vector of rotation) shows more variation due to the fact that the nanostructure diffuses, thereby changing the distance between the nanostructure and the interface. A supplementary video of the nanostructure movement (video S4) is available online.

Specifically, we showed previously that it is possible to use this material as propellers by actuating the micro- and nanostructures with rotating magnetic fields^16^. An example of a rotating magnetic field is shown in Figure 1b. The random shape of the particles makes them sufficiently asymmetric, which leads to a hydrodynamic coupling between rotation and translation (Figure 2b). The propellers move approximately parallel to the vector of rotation of the magnetic field (Figure 2a). Deviations from a straight line are apparent and due to diffusion, which is superimposed on the linear movement of the propeller^16^. Nevertheless, the movement of the propellers can be controlled and the particles follow directional changes of the actuating field (Figure 3). We measure an average propulsion speed of 3.8 μm × s^−1^ when actuating structures at 20 Hz. The structures had an average size of around 6.3 μm and were used as synthesized, without selection. This propulsion speed is comparable to that of designed propellers^27^. The relationship between actuating frequency and propulsion speed can be understood based on a simple torque-balance model^16^.

Rolling motion is achieved also by the application of rotating magnetic fields, like those used for propulsion. However, in this case, the structures need to be close to an interface, which is easily achieved by sedimentation. The movement due to the rolling effect (Figure 2d) is perpendicular to the vector of rotation of the magnetic field (Figure 2c). Not surprisingly any structure which is forced to rotate close to a solid interface starts to roll^18^. Depending on the strength of the hydrodynamic coupling between rotation and translation, the direction of movement will have a smaller or larger component parallel to the vector of rotation of the magnetic field^19^. Despite their random shapes, sufficiently large structures move in a consistent way, meaning that the motion proceeds along a line and at a constant speed, which depends on the frequency of actuation^18^,^35^. In contrast to propulsion, diffusive motion is not simply superimposed on the rolling motion, since the rolling speed changes, depending on the distance between the roller and the interface. Small structures for which diffusive motion away from the interface is not negligible can thus display a non-constant rolling speed (see Figure 3b). When actuated with a 20 Hz rotating field the average rolling speed is around 7.2 μm × s^−1^ (at a mean size of around 6.3 μm). Again the structures were used as synthesized without selection and the observed rolling speeds are comparable to those of previously published rollers^27^.

**Figure 4:**
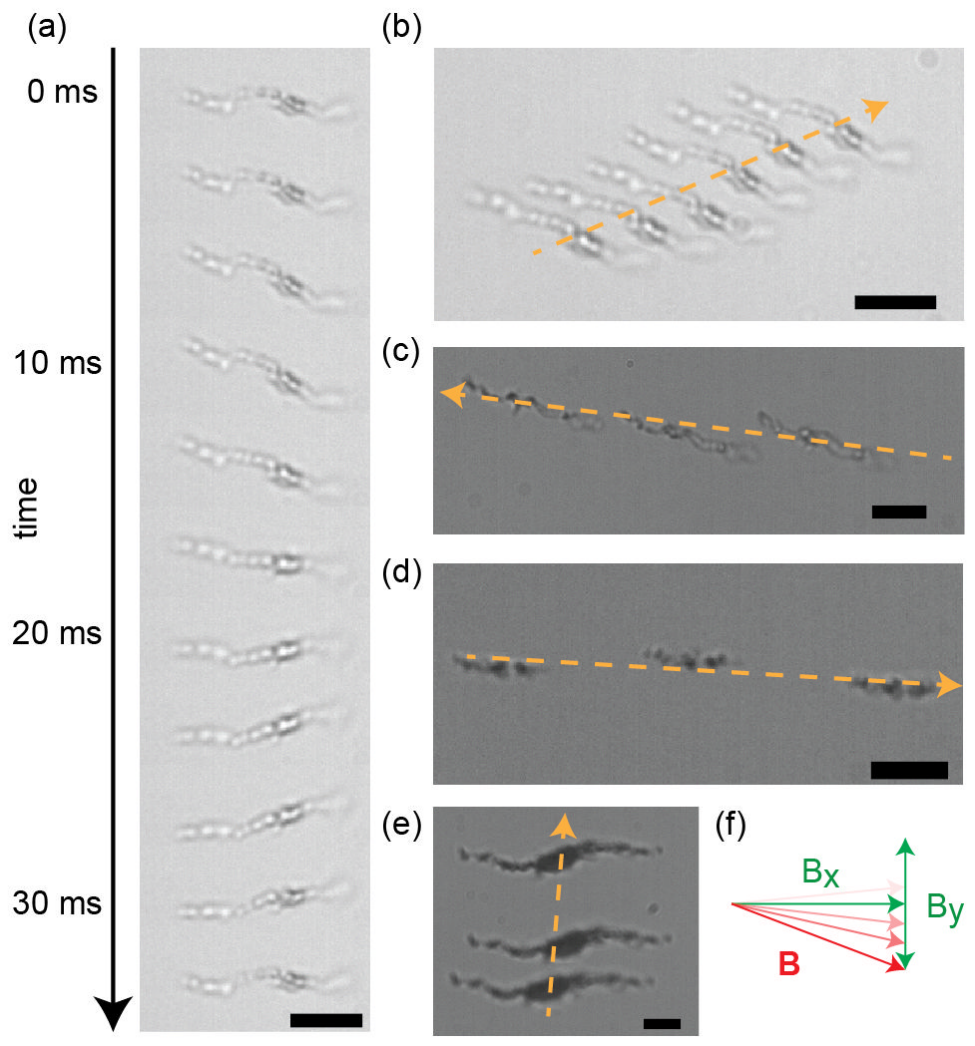
Self-assembled swimmers form and start moving under the influence of a time-reversible actuating field. The x-component (horizontal) of the field has constant field strength and the y-component (vertical) is sinusoidal with a frequency of 30 Hz. (a) Sequential shape and orientation changes of a self-assembled swimmer. Images are cut from a video recorded at 600 fps. (b) Time-lapse image of the movement of the self-assembled swimmer from (a). The field strengths are 2 mT and 1 mT along the x and y directions respectively. Images are overlaid in 1 s intervals. (c) A different self-assembled swimmer is moving in the same field as (b). Images are overlaid in 7 s intervals. (d) Another self-assembled swimmer is actuated by a different field. The field strengths are 1 mT and 2 mT along the x and y directions respectively. Images are overlaid in 6.4 s intervals. (e) Another self-assembled swimmer is moved by the same field as (d). Images are overlaid in 10 s intervals. (f) Schematic representation of the applied fields. The dashed orange arrows indicate the directions of the movements. The scale bars are 5 μm.

**Figure 5:**
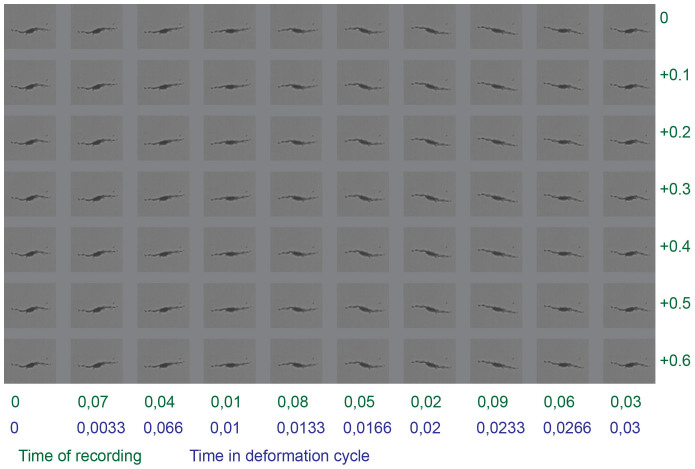
Cyclic shape deformation of the self-assembled swimmer presented in Figure 4e. The images were recorded at times given in green (column time + row time). All times are in seconds. The images are rearranged in the order in which they appear in the cyclic shape deformation (in blue), based on the assumption that the frequency of the cyclic shape deformation is 30 Hz. The rearrangement is necessary because the magnetic actuating field is oscillating with 30 Hz, whereas the video was recorded with 100 frames per second. The width of the single images is 36 μm.

Finally, swimming motion is achieved using the same material, by actuating a self-assembled flexible chain-like structure with a magnetic field that would induce reciprocal motion in a rigid structure. An example of such a magnetic field is shown in Figure 1a. The individual aggregates used for propelling and rolling are too stiff to be bent by magnetic actuation and hydrodynamic forces. Individual aggregates thus cannot be directly used as swimmers. Several aggregates are, however, able to swim when being assembled into a chain-like structure by applying a constant magnetic field first. Since the individual aggregates in the chain-like assembly have different shapes and assemble into an asymmetrically shaped swimmer, symmetry breaking is immediately achieved. We used a high-speed camera to image the deformations induced in an exemplary chain-like assembly (Figure 4a). The chain-like assembly goes through these deformations repeatedly (see Figure 5), which leads to translatory movement along a linear trajectory (Figure 4b). Due to the asymmetry of the assembled chain-like structure, the deformation cycle is non-reciprocal, leading to a net motion (swimming).

The observed swimmers were moving close to a glass interface. Therefore, the swimming speed might be different if a swimmer would move in bulk low Reynolds number liquid instead, due to the altered hydrodynamic situation. However, as the surface does not break the 2D translational symmetry in the plane parallel to the surface in which the swimmers are moving, the non-reciprocal deformation cycle must be behind the observed swimming motion. This means that the swimmers would also be able to move in bulk liquid, although they would probably move at a different speed and sediment under the influence of gravity in addition to the swimming motion.

Typically such swimming movements have been described as a bending wave traveling along a filament^22^,^36^. In this framework, it would be expected that the structure moves parallel to its long axis. We find, however, that different chain-like assemblies can move in seemingly arbitrary directions while being actuated by the same magnetic field. Some examples are given in Figure 4. The self-assembled swimmers (Figure 4b–e) move with 3.5 μm × s^−1^, 2.1 μm × s^−1^, 1.8 μm × s^−1^ and 1.6 μm × s^−1^ respectively. A particular chain-like assembly will move along a linear trajectory persistently, but the direction of movement can be different for different assemblies. This shows that the chain-like assemblies are not moving erratically, but are continuously repeating a particular set of deformations.

The different chain-like assemblies we obtain demonstrate for the first time that chain-like assemblies do not necessarily move in the direction of their long-axis. To our knowledge, all previously published microscopic flexible swimmers moved along their long axis^22^,^23^,^24^,^25^,^26^,^27^. From a theoretical standpoint, it has been known for a long time that the direction of movement of a swimmer is determined by the cyclic deformation it undergoes and that this movement does not need to be parallel to the long axis of a chain-like swimmer^28^,^29^,^31^,^37^,^38^. An example is the deformation cycle of the self-assembled swimmer in Figure 4e, which is displayed in Figure 5. The random assemblies that form the swimmers in this study offer the possibility to expand the range of swimmer designs and observe also deformation cycles that lead to movement perpendicular to the swimmers long axis.

**Figure 6:**
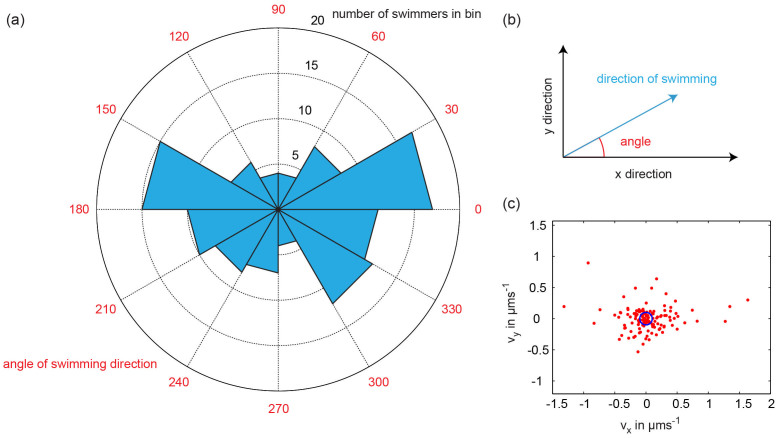
Angular distribution of swimming direction. The speeds of 128 self-assembled swimmers were measured to construct this diagram. All self-assembled swimmers were actuated by a constant magnetic field of 2 mT strength in x direction and a 30 Hz sinusoidal field with amplitude 1 mT in y direction. (a) Rose diagram of swimming direction angles. (b) The swimming direction angle is defined as the angle between the direction of swimming and the x-axis, which is the long axis of the self-assembled swimmers. (c) The x components of the measured swimming speeds are plotted against the y components. The blue circle in the middle is centered around the origin and has radius 0.1 μms^−1^. Swimmers with speeds below this threshold of 0.1 μms^−1^ were not included in the rose diagram of panel (a), in order to exclude swimming directions that might be influenced by Brownian motion.

Next, we observed the movement of further 128 self-assembled swimmers to study the motion of self-assembled swimmers in more detail. All swimmers were actuated by a constant field of 2 mT strength in the x direction and a 1 mT sinusoidal field with frequency 30 Hz in the y direction. The speeds of the swimmers are displayed in Figure 6c. The mean values of the x component (〈*v_x_*〉 = 0.03 μm × s^−1^) and the y component (〈*v_y_*〉 = −7 × 10^−4^ μm × s^−1^) of the swimming speed are both close to zero for symmetry reasons. The mean values of the absolute values are 〈|*v_x_*|〉 = 0.24 μm × s^−1^ and 〈|*v_y_*|〉 = 0.14 μm × s^−1^ respectively. We restrained the measurement of the angular distribution of swimming directions by excluding the swimmers that moved slower than 0.1 μm × s^−1^, in order to avoid swimming directions that might have been influenced by Brownian motion. The resulting angular histogram is displayed in Figure 6a. The speeds of all 128 self-assembled swimmers are displayed in Figure 6c. The observed distribution of swimming directions supports the claim that swimmers can move in seemingly arbitrary directions. There is a preference to move along the long axis of the swimmer, but a significant number of swimmers also move approximately perpendicular to the long axis. The preference to move along the long axis of the swimmer might possibly be related to differences in hydrodynamic drag, although further theoretical studies would be needed to determine if this is in fact the case. For a thin rod in bulk low Reynolds number liquid, the drag coefficient for movement perpendicular to the long axis is indeed twice as high as that for movement along the long axis^39^.

In general, the magnetic particles used in this study have several properties, which seem to be essential for the reported reproduction of all three types of magnetic actuation. Firstly, the material has a sufficiently high volume magnetization to be actuated by weak external magnetic fields (1–2 mT), but the magnetization is not so high as to lead to irreversible aggregation due to inter-particle forces. Secondly, the carbon coating fixes the structures into aggregates of varied shapes, which can resist hydrodynamic shear and lead to the hydrodynamic coupling between rotation and translation. The carbon coating also passivates individual structures, so that the surfaces of different aggregates repel each other and aggregation due to van-der-Waals or electrostatic interactions is avoided. This is particularly important for the self-assembly of chain-like swimmers. The chain-like assemblies are indeed held together by magnetic interactions, but the short range repulsion that avoids aggregation keeps the assemblies flexible, so that cyclic deformations can lead to swimming.

In summary, while other groups have shown that all three types of magnetic actuation for microscopic devices in liquid can be realized with structures specifically designed for a particular type of actuation, we show here that this can be achieved as well using a single and cheaply producible type of nanostructured magnetic material. Therefore, new designs for magnetic actuators would need to demonstrate that they are in some way superior to random structures, in order to justify the design and synthetic effort. Chain-like assemblies of our nanostructures not only reproduce magnetically actuated swimming but also show experimentally that the swimming direction of a chain-like assembly does not need to be parallel to the long axis of the assembly. Our findings demonstrate that precise control over the shape of magnetic actuators is not a prerequisite for obtaining movement and that in contrast, variety of shapes can lead to versatile possibilities for actuation. The shape of micro- and nanorobots could thus be tailored to other requirements (e.g. manipulation and sensing capabilities or ease of fabrication) without compromising the ability to power and steer such devices with external magnetic fields.

## Methods

The synthesis method for the production of the carbon coated magnetic nanostructures has been described previously^16^. Magnetic iron oxide nanoparticles (NanoArc iron(III) oxide, Alfa Aesar) are suspended in a glucose solution and heated to 180°C for 24 h. The iron oxide catalyses the thermal decomposition of the glucose, which leads to the deposition of a carbon layer on the iron oxide nanoparticles^40^,^41^,^42^. In this way, aggregates of magnetic nanoparticles are structurally fixed. Reaction products were washed several times with ethanol and de-ionized water, using magnetic separation, and used without further processing.

Optical microscopy was performed on a custom-made microscope based on a slotted aluminum baseplate^16^,^43^. The microscope was equipped with custom made triaxial Helmholtz coils with controller (C-SpinCoil-XYZ, Micro Magnetics Inc.), a high speed camera (CR3000x2, Optronis) and an illumination source (pE-100, 635 nm, CoolLED Ltd.). The sample is held in the centre of the magnetic setup by a custom-made aluminum sample holder secured on a xyz motorized translation stage (PT3/M-Z8, Thorlabs), which was controlled by supplier software (apt user application, Thorlabs). Experiments were performed in glass capillaries (0.2 × 2 × 50 mm, Vitrotubes, Vitrocom).

Chain-like assemblies of particles were created by applying a constant homogenous field of 1 or 2 mT. This aligned the individual particles and over the course of seconds to minutes the particles assembled into chain-like structures. Then a sinusoidal field of field strength 1 or 2 mT was applied in the imaging plane, perpendicular and in addition to the constant field that aligned the particles.

## Supplementary Material

Supplementary Information supplementary file

Supplementary Information video S1

Supplementary Information video S2

Supplementary Information video S3

Supplementary Information video S4
