# Supplementary material for: The triathlon of magnetic actuation: Rolling, propelling, swimming with a single magnetic material
Source: Sci Rep. 2015 Mar 20;5:9364. doi: 10.1038/srep09364 (PMC4366818; doi:10.1038/srep09364)
Supplement: Supplementary Information — supplementary file [file srep09364-s1.docx]

The triathlon of magnetic actuation: Rolling, propelling, swimming with a single magnetic material

Peter J. Vach and Damien Faivre

Supplementary Material

**Description of Supplementary Videos:**

Video S1: Video of propeller motion. The actuating field is first visualized followed by a video of propeller motion induced by the actuating field. The video of propeller motion is shown four times slower than real time and corresponds to Figure 2 a.

Video S2: Video of rolling motion. The actuating field is first visualized followed by a video of rolling motion induced by the actuating field. The video of rolling motion is shown four times slower than real time and corresponds to Figure 2 c.

Video S3: Videos of self-assembled swimmers. The actuating field is first visualized followed by the motion of a self-assembled by the same actuating field. The factor by which a video is slowed down with respect to real time is indicated as a text overlay in the video. Self-assembled swimmers displayed in Figure 4 a, b, d and e appear in the video.

Video S4: Video of magnetically actuated nanostructure changing the direction of motion. This video is analyzed in Figure 3.

**Supplementary Figures**


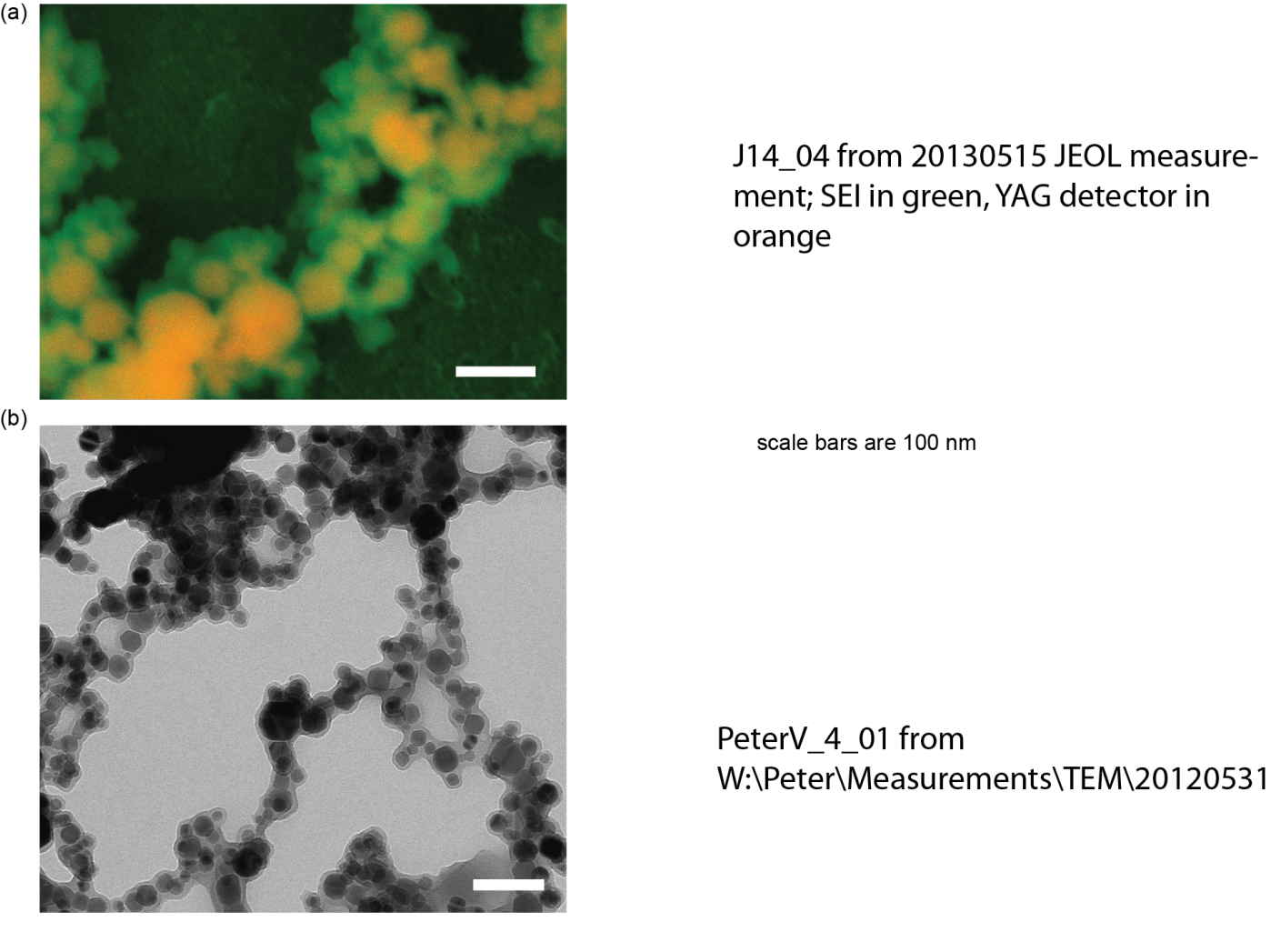


Figure S1: Characterization of the as-synthesized material by electron microscopy. (a) Overlay image of back-scattered signal (orange) and secondary electron signal (green). The iron oxide particles give a strong back-scattered signal, while the carbon coating can be seen in green. (b) Transmission electron microscopy image of the material. The iron oxide particles can be clearly seen inside the carbon coating. Scale bars are 100 nm.


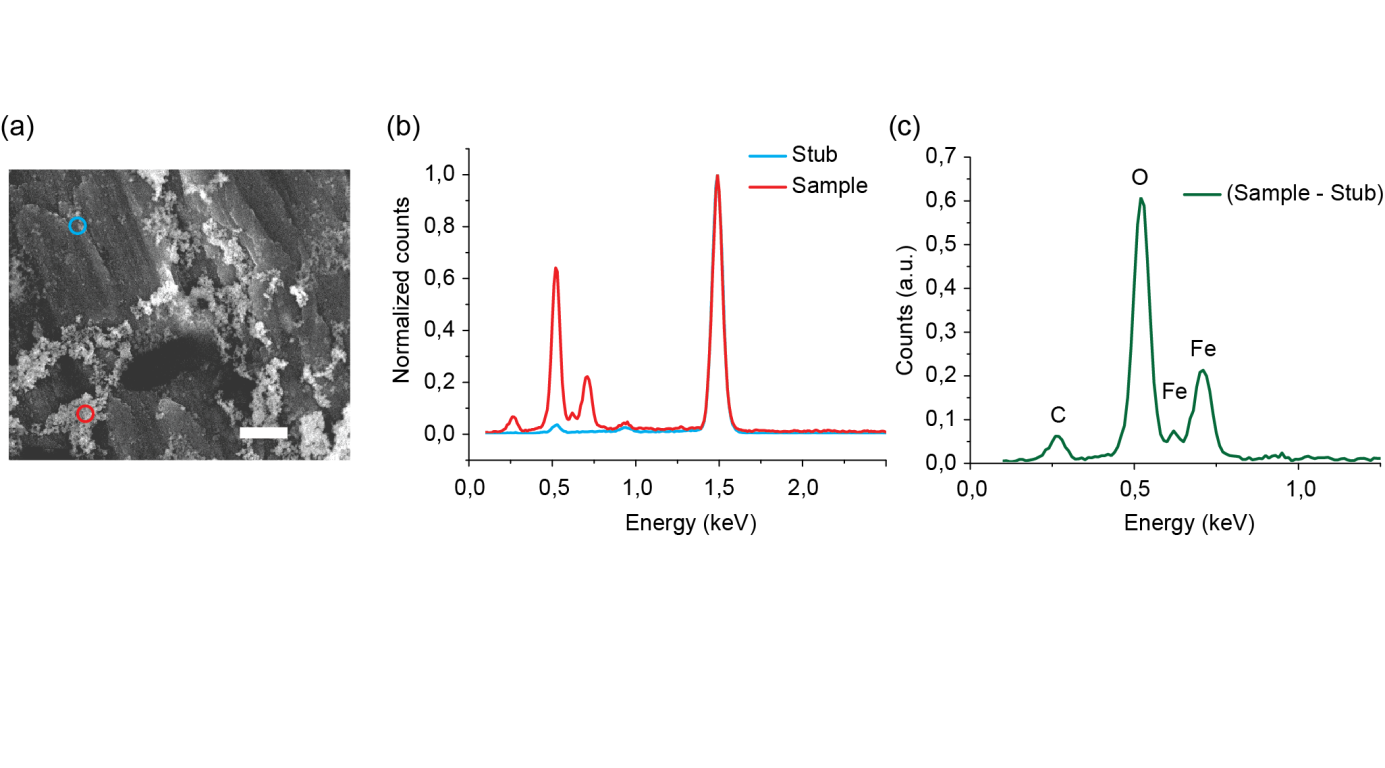


Figure S2: (a) Secondary electron image of as-synthesized nanostructures dried onto an aluminum stub in a 100 Hz, 1mT rotating magnetic field. The blue circle marks the spot where an EDX measurement was performed on the stub. The red circle marks the spot where an EDX measurement was performed on the synthesized nanostructures. The scale bar is 1 µm. (b) The two EDX spectra of the stub (blue) and the nanostructures (red) were normalized by their maximum value (peak due to aluminum). (c) The difference between the normalized stub spectrum and the normalized nanostructure spectrum is plotted.


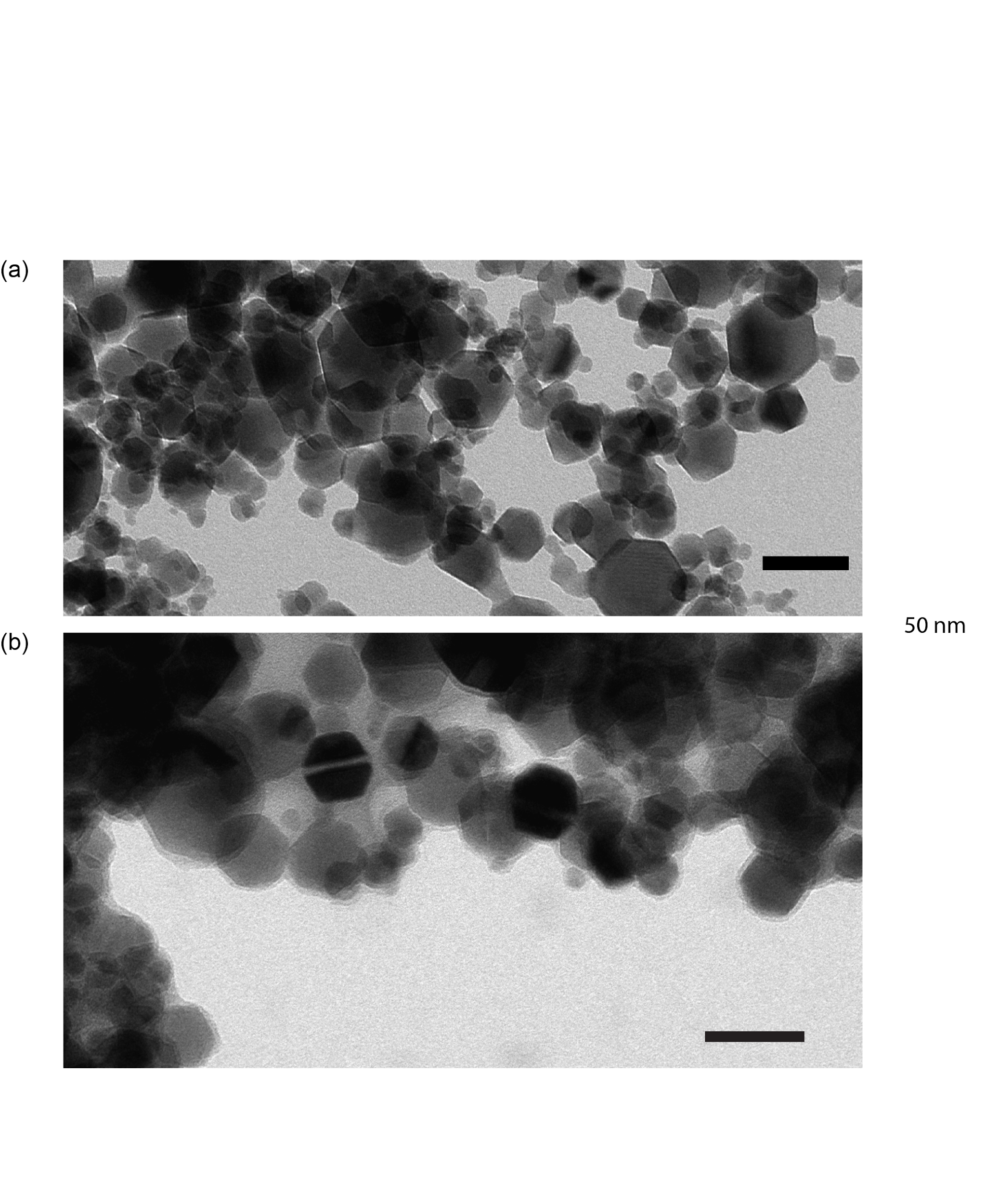


Figure S3: (a) Transmission electron microscopy image of untreated NanoArc iron(III) oxide nanoparticles. For sample preparation, particles were dried onto a carbon film suspended on a copper grid after dispersing the particles in deionized water. (b) Transmission electron microscopy image of NanoArc iron(III) oxide nanoparticles after HTC treatment as described in the main text. The carbon coating is visible, especially in comparison with the untreated nanoparticles in panel (a). Scale bars are 50 nm.


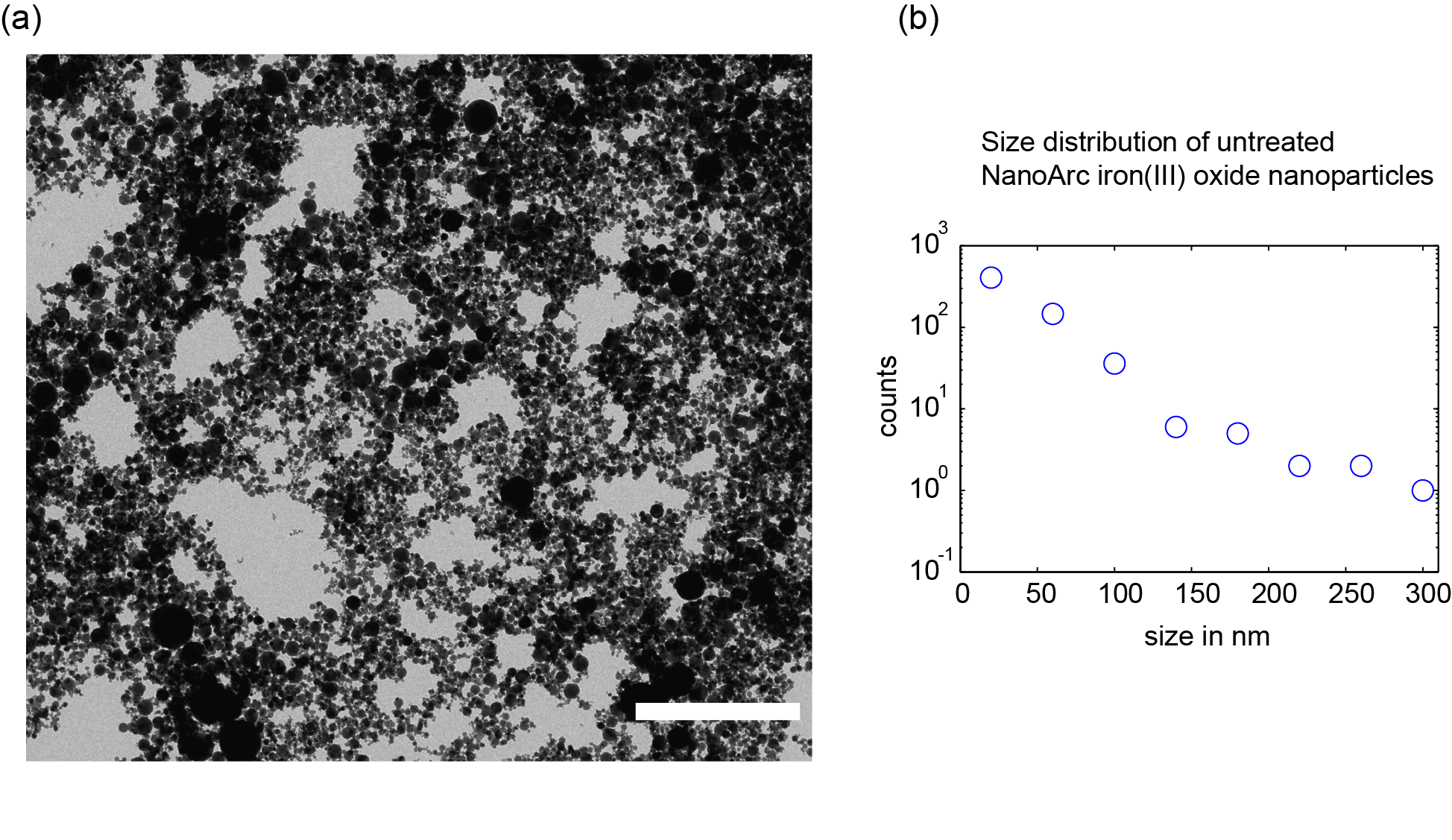


Figure S4: (a) Overview TEM image of untreated NanoArc iron(III) oxide nanoparticles. Scale bar is 1 µm. (b) Approximate size distribution of the nanoparticles shown in (a). The size distribution is based on 605 manual size measurements (using ImageJ) of a subset of the imaged nanoparticles. This is not a very accurate method of measuring the nanoparticle size distribution but it is sufficiently accurate to demonstrate the broadness of the size distribution.

**Additional methods**

Electron microscopy

The nanostructure of the synthesized iron-oxide carbon hybrid material was investigated using electron microscopy. Scanning electron micrographs were obtained either with a Jeol JSM7500F. For transmission electron microscopy, samples were prepared by drying a certain volume of suspended nanostructures onto a carbon coated grid. Imaging was performed on a Zeiss EM 912 Omega with 120 kV acceleration voltage.

EDX

The elemental composition of the synthesized nanostructures was investigated using energy-dispersive x-ray spectroscopy (EDX). Measurements were performed at 8kV and the spectra were acquired using an Oxford Inca Energy Dispersive Spectroscopy System equipped with an X-Max silicon drift detector.
